# Dynamic chaotic gravitational search algorithm-based kinetic parameter estimation of hepatocellular carcinoma on ^18^F-FDG PET/CT

**DOI:** 10.1186/s12880-022-00742-4

**Published:** 2022-02-06

**Authors:** Jianfeng He, Tao Wang, Yongjin Li, Yinglei Deng, Shaobo Wang

**Affiliations:** 1 Faculty of Information Engineering and Automation, Kunming University of Science and Technology, Yunnan Key Laboratory of Artificial Intelligence, Kunming, 650500 Yunnan China; 2 PET/CT Center, Affiliated Hospital of Kunming University of Science and Technology, First People’s Hospital of Yunnan, Kunming, 650031 China

**Keywords:** Kinetic models, PET/CT, Hepatocellular carcinoma, Gravitational search algorithm

## Abstract

**Background:**

Kinetic parameters estimated with dynamic ^18^F-FDG PET/CT can help to characterize hepatocellular carcinoma (HCC). We aim to evaluate the feasibility of the gravitational search algorithm (GSA) for kinetic parameter estimation and to propose a dynamic chaotic gravitational search algorithm (DCGSA) to enhance parameter estimation.

**Methods:**

Five-minute dynamic PET/CT data of 20 HCCs were prospectively enrolled, and the kinetic parameters *k*_1_ ~ *k*_4_ and the hepatic arterial perfusion index (*HPI*) were estimated with a dual-input three-compartment model based on nonlinear least squares (NLLS), GSA and DCGSA.

**Results:**

The results showed that there were significant differences between the HCCs and background liver tissues for *k*_1_, *k*_4_ and the *HPI* of NLLS; *k*_1_, *k*_3_, *k*_4_ and the *HPI* of GSA; and *k*_1_, *k*_2_, *k*_3_, *k*_4_ and the *HPI* of DCGSA. DCGSA had a higher diagnostic performance for *k*_3_ than NLLS and GSA.

**Conclusions:**

GSA enables accurate estimation of the kinetic parameters of dynamic PET/CT in the diagnosis of HCC, and DCGSA can enhance the diagnostic performance.

## Background

Hepatocellular carcinoma (HCC) is the third leading cause of cancer-related death, with insignificant clinical manifestations and concealed symptoms at the initial stage of the disease [1, 2]. Conventional medical imaging techniques such as computed tomography (CT) and magnetic resonance imaging (MRI) are often used for the initial examination in clinical practice. However, they can only generate structural images and lack tumor metabolic information [3]. Positron emission tomography (PET)/CT has emerged as a noninvasive functional imaging method that allows assessment of metabolic function in tumors by injecting glucose analogs as radiotracers [4].

Although static ^18^F-FDG PET imaging obtains several parameters, such as standard uptake values (SUV), metabolic tumor volume and total lesion glycolysis, it is insufficient to describe the metabolic processes of ^18^F-FDG [5]. Dynamic PET/CT imaging can track the distribution of ^18^F-FDG in tissues and derive some kinetic parameters that accurately describe the cellular metabolic processes of ^18^F-FDG to enhance diagnosis and therapy in various diseases, and compartmental modeling is routinely applied to estimate kinetic parameters [6, 7]. Sarkar et al. [8] demonstrated that dynamic ^18^F-FDG PET with tracer kinetic modeling has the potential to diagnose nonalcoholic steatohepatitis. Wang et al. [9] found that dynamic ^18^F-FDG PET with optimization-derived blood input function kinetic modeling can effectively distinguish liver lesions. Considering 60-min or more dynamic PET/CT is not easily available in routine clinical settings. Samimi et al. [10] reported that 5-min dynamic PET / CT plus a static PET/CT enables accurate and robust estimation of kinetic parameters in patients with liver metastases. Our previous studies [11, 12] also demonstrated that 5-min dynamic ^18^F-FDG PET/CT can provide blood flow and metabolic information that enhances the detection of HCC lesions [11], and derived perfusion and early-uptake PET/CT are feasible for diagnosing HCC and provide added functional parameters to enhance diagnostic performance [12].

Nonlinear least squares (NLLS) is commonly used to estimate compartment model parameters [10, 12–14], and its essence is to minimize the sum of squared errors of the measured and estimated values; however, NLLS easily falls into the local optimum in the process of parameter estimation, and the set initial value has a great influence on the results [15]. The gravitational search algorithm (GSA) is a swarm intelligent optimization algorithm that is based on Newtonian gravity and has a strong searching ability [16]. It is more stochastic, does not require initial values, and can perform better parameter estimation compared to NLLS; however, whether GSA can function well to estimate compartment model parameters with dynamic ^18^F-FDG PET/CT is unclear. Additionally, a dynamic chaotic gravitational search algorithm (DCGSA) is proposed to improve the exploration ability and global search ability to enhance parameter estimation. Therefore, this study evaluated the role of the compartmental parameters of 5-min ^18^F-FDG PET/CT estimated by NLLS, GSA and DCGSA for distinguishing HCC from background liver tissue.

## Methods

### Patients

This study was approved by the Institutional Review Committee (IRB) of the First People’s Hospital of Yun-nan Province (No. 2017YYLH035), informed consent was obtained from all the patients, and all the methods were performed in accordance with the Declaration of Helsinki.

We recruited 28 patients with clinically suspected HCC, and a 5-min dynamic PET/CT scan was added before conventional PET/CT. Ten patients were excluded because they had a non-HCC pathological diagnosis (n = 5), lacked a pathological diagnosis (n = 3), and had suboptimal imaging quality (n = 2).

Eighteen patients (17 males and 1 female) who had pathologically confirmed HCC were finally included in this study. Sixteen patients had a single lesion, and two patients had two lesions. A total of 20 HCC tumors that were confirmed by surgery (n = 14) or biopsy (n = 6) were used in this study, and the long axis of these tumors was 1.9–15.0 cm (average 6.5 ± 3.6).

### Dynamic PET/CT

All examinations were performed using a Philips Ingenuity TF PET/CT scanner (Cleveland, OH, USA), and a Philips IntelliSpace Portal v7.0.4.20175 was used for postprocessing. In summary, after the patients had fasted for at least 6 h, blood glucose was verified. A low-dose liver CT scan (120 kV, 100 mAs) was performed for attenuation correction and image fusion. A 5-min dynamic PET scan was performed over the liver region after intravenous administration of 5.5 MBq/kg ^18^F-FDG. Dynamic PET data were divided into 16 frames using the following sampling schedule: 12 frames of 5 s and 4 frames of 60 s each. Dynamic PET images were then reconstructed using the standard ordered subsets expectation maximization (OSEM) algorithm.

**Fig. 1:**
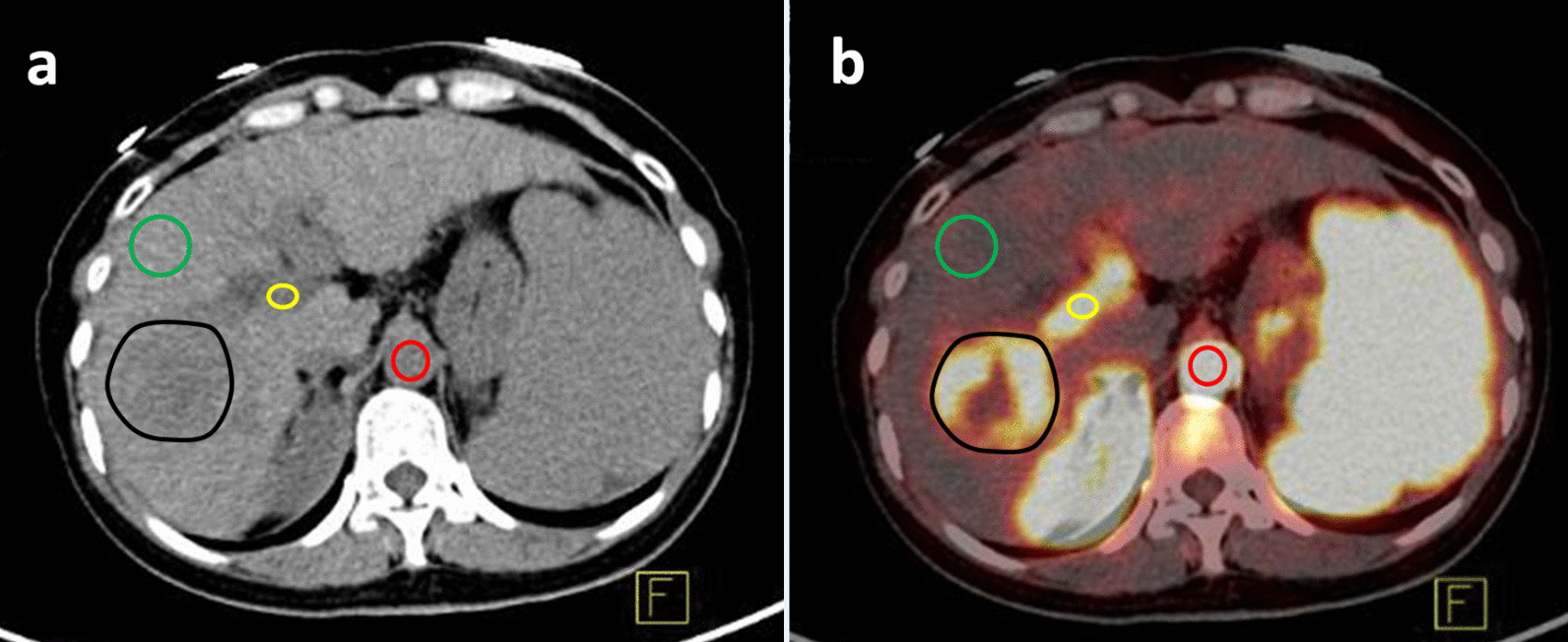
Region of interest drawn in dynamic PET/CT. **a** CT image, **b** PET/CT fusion image. The HCC is shown in the black circle, background liver tissue is shown in the green circle, the portal vein is shown in the red circle, and the aorta is shown in the yellow circle. Blood ^18^F-FDG enters the aorta, portal vein, spleen and HCC

Regions of interest (ROIs) were drawn manually in the CT images of each patient, including the HCC, background liver tissue, aorta and portal vein (the extrahepatic portal vein rather than the intrahepatic portal vein), with manual slice-by-slice adjustment. An ROI was copied to the PET/CT images after image fusion, and time activity curves (TACs) consisting of the maximum SUV (SUVmax) extracted from each frame were generated. Figure 1 shows the ROIs drawn on a transaxial dynamic PET/CT image of a patient with HCC.

### Kinetic modeling

**Fig. 2:**
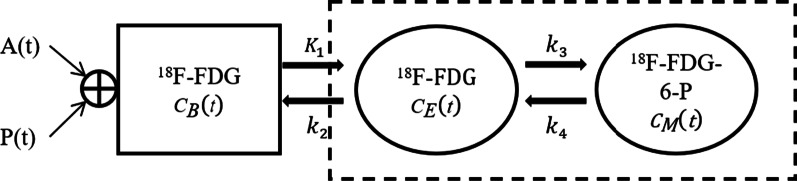
A dual-input three-compartment model

A dual-input three-compartment model was used to assess the steady-state hepatic metabolism of ^18^F-FDG, as shown in Fig. 2 [17, 18].

In Fig. 2, *k*_1_ (ml/min/ml) represents the rate constant of ^18^F-FDG from the blood to the liver tissue, and *k*_2_ represents the clearance rate back to the blood. *k*_3_ is the rate constant of further phosphorylation of ^18^F-FDG to ^18^F-FDG-6-phosphate and *k*_4_ is the dephosphorylation rate of phosphatase. *C*_*B*_(*t*) represents the ^18^F-FDG concentration in the blood:

1 $${C}_{B}\left(t\right)=HPI\times A\left(t\right)+\left(1-HPI\right)\times P\left(\mathrm{t}\right),$$

where *A*(*t*) represents the ^18^F-FDG concentration in the hepatic artery, and *P*(*t*) represents the ^18^F-FDG concentration in the portal vein. The *HPI* represents the hepatic artery perfusion index (the ratio of arterial blood volume to total blood volume). *C*_*M*_(*t*) represents the free-state ^18^F-FDG concentration and the metabolized ^18^F-FDG-6-phosphate concentration in the liver tissue compartment. *C*_*T*_(*t*) represents the curve of the tracer concentration in the tissue measured from the PET image over time and is the output function of the kinetic model:

2 $${C}_{T}\left(\mathrm{t}\right)=\frac{{K}_{1}}{{\alpha }_{2}-{\alpha }_{1}}\times \left[\left({k}_{3}+{k}_{4}-{\alpha }_{1}\right){e}^{-{\alpha }_{1}t}+\left({\alpha }_{2}-{k}_{3}-{k}_{4}\right){e}^{-{\alpha }_{2}t}\right]\otimes{C}_{B}\left(t\right)$$

where ${\alpha}_{2} \, {\text{and}} \, {\alpha}_{1}$ can be described as follows:

3 $${\alpha }_{1}=\frac{{k}_{2}+{k}_{3}+{k}_{4}-\sqrt{{({k}_{2}+{k}_{3}+{k}_{4})}^{2}-4{k}_{2}\times {k}_{4}}}{2}$$

4 $${\alpha }_{2}=\frac{{k}_{2}+{k}_{3}+{k}_{4}+\sqrt{{({k}_{2}+{k}_{3}+{k}_{4})}^{2}-4{k}_{2}\times {k}_{4}}}{2}$$

### Estimation of the kinetic parameters

This study proposes an improved algorithm, the DCGSA, for the estimation of liver kinetic parameters. In the GSA, a solution space about the objective function is initialized randomly, with each individual in the space as one feasible solution in the objective function. With constant movement, individuals will move toward the most mass, which is the optimal solution of the search space [16]. The convergence speed of the GSA is faster, which makes it fall into a local optimum without global search [19].

**Fig. 3:**
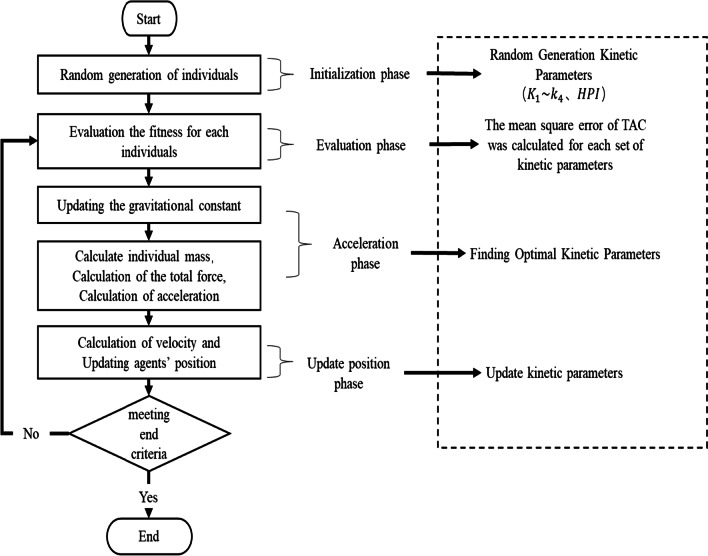
Flowchart of the DCGSA

In the DCGSA, the dynamic adjustment strategy is introduced for the gravitational constant of GSA to improve the algorithm exploration capacity and mining capacity. At the same time, inertia weights and chaotic sequences are added to the particle speed update process to avoid falling into a local optimum and to improve the global search ability. The overall flow of the DCGSA is shown in Fig. 3. The DCGSA can be simply divided into four parts: the initialization phase, the evaluation phase, the acceleration phase, and the update position phase.

### Initialization phase

To start the search process for the DCGSA, the initial population of *N* individuals is randomly generated in the search space, which represents a set of model parameters (*k*_1_, *k*_2_, *k*_3_, *k*_4_ and the *HPI*). The position of each particle is represented by the following:

5 $${X}_{i} =\left({x}_{i}^{1}, {x}_{i}^{2},\cdots ,{x}_{i}^{d},\cdots ,{x}_{i}^{D}\right)\quad \text{for}\,\, i=1,\cdots ,N$$

where ${x}_{i}^{D}$ is the position of the *i*th individual in the D dimension.

### Evaluation phase

The individual is evaluated according to the fitness function, defined as the sum of the squared errors squared errors of the experimental data and the fitted data:

6 $${fit}_{i}\left(t\right)= {\sum }_{i=1}^{N}{\left( {{C}_{T}}^{^{\prime}}\left(t\right)- {C}_{i}^{m}\left(t\right) \right)}^{2},$$

where *N* is the number of individuals, *i* is the index, ${{C}}{(}{{t}}{)}$ is the actual concentration of ^18^F-FDG in the obtained tissue, and ${{C}}_{{i}}^{{m}}{(}{{t}}{)}$ is the estimated concentration of ^18^F-FDG in the obtained tissue. After that, the individual continuously updates its inertial mass ${M}_{i}\left(t\right)$ during movement by the following equations, that is, to find the best model parameters in the search space:

7 $${{m}}_{{i}}\left({{t}}\right)= \frac{{{fi}}{{t}}_{{i}}\left({{t}}\right)- \text{worst}\left({{t}}\right)}{{\text{best}}\left({{t}}\right)-\text{worst}\left({{t}}\right)}$$

8 $${{M}}_{{i}}\left({{t}}\right)= \frac{{{m}}_{{i}}{(t)}}{{\sum }_{{j=1}}^{{N}}{{{m}}}_{{j}}{(t)}},$$

where *best(t)* and *worst(t)* are the best and worst fitness, respectively:

9 $$\mathrm{best}\left(t\right)= \underset{\mathrm{j}\in 1,\cdots ,\mathrm{N}}{\mathrm{min}}{\mathrm{fit}}_{\mathrm{j}}\left(\mathrm{t}\right)$$

10 $$\mathrm{worst}\left(t\right)= \underset{\mathrm{j}\in \{1,\cdots ,\mathrm{N}\}}{\mathrm{max}}{\mathrm{fit}}_{\mathrm{j}}(\mathrm{t})$$

### Acceleration phase

The individual will be subjected to the force of other individuals in the search space. Based on the law of universal gravitation, the force ${\text{F}}_{\text{ij}}^{\text{d}}\left({\text{t}}\right)$ of individual *i* by individual *j* is:

11 $${F}_{ij}^{d}\left(t\right)=G\left(t\right)\frac{{M}_{i}\left(t\right)\times {M}_{j}\left(t\right)}{{R}_{ij}\left(t\right)+ \varepsilon }\left({x}_{i}^{d}\left(t\right) - {x}_{j}^{d}\left(t\right)\right)$$

where *M*_*i*_(*t*) and *M*_*j*_(*t*) are the inertial masses of individuals *i* and *j* at time *t*, respectively, that is, the TACs calculated by a set of kinetic parameters. $\epsilon$ is a small value to prevent errors. *R*_*ij*_*(t)* is the Euclidean distance between individuals *i* and *j*. *G*(*t*) is a gravitational constant that decreases with time *t* and is described by Eq. (12); it can affect the force and acceleration of the individual:

12 $$G\left(t\right)={G}_{0} {e}^{-\alpha \frac{t}{T}}$$

where ${\text{G}}_{0}$ is an initial value, α is a constant, *t* is the current number of iterations, and *T* is the maximum number of iterations. The traditional gravitational constant was not fully explored in the early stage of iteration, and fell into a local optimum [20]. Lei et al. [21] introduced a self-adaptive gravitational constant, and Seyedali et al. [22] used chaotic mapping to adjust the gravitational constant.

Therefore, this paper proposes a new gravitational constant expression, introducing an improved dynamic adjustment strategy, which contains a random variable as follows:

13 $${G}{{^{\prime}}\left(t\right)}={G}_{0} {e}^{-\alpha \frac{t}{{T}^{1.5}} \times \left({rand}_{t}+\frac{t}{T}\right)},$$

where ${\text{rand}}_{\text{t}}$ is a random number in (0,1). $G{^{\prime}}\left(t\right)$ adopts a large step and long movement in the early stage of iteration to increase the particle exploration ability and enough time for optimization. Random variables can abruptly change the gravitational constant during iterations, improving the ability of particles to jump out of the local optimum. The process of moving in small steps is adopted in the later stage of iteration, which effectively avoids the premature convergence of particles and improves the mining capacity.

The resultant force of each particle is calculated as follows:

14 $${F}_{i}^{d}\left(t\right)=\sum_{j\in Kbest,j\ne i}{rand}_{j}{F}_{ij}^{d}\left(t\right)$$

where ${\text{Kbest}}$ decreases with the number of iterations, the initial value is *N*, and ${\text{rand}}_{\text{j}}$ is a random number between the interval (0, 1). The acceleration of particle *i* at time *t* is calculated as follows:

15 $${a}_{i}^{d}\left(t\right)= \frac{{F}_{i}^{d}(t)}{{M}_{i}(t)}$$

### Update position phase

In each iteration, the particles update their velocity and position as follows:

16 $${v}_{i}^{d}\left(t+1\right)= {rand}_{i} \times {v}_{i}^{d}\left(t\right)+ {a}_{i}^{d}\left(t\right)$$

17 $${x}_{i}^{d}\left(t+1\right)= {x}_{i}^{d}\left(t\right)+{v}_{i}^{d}(t+1)$$

where ${\text{rand}}_{\text{i}}$ is a uniformly distributed random number between the interval (0, 1). However, some particles move too fast in the moving process, thus flying out of the solution space. Li et al. [23] introduced inertia weight instead of random variables to restrict the particle velocity. Gao et al. [24] replaced random variables with chaotic sequences. Therefore, this paper adds inertia weight and chaotic sequence to the particle speed updating process to further limit the particle speed:

18 $${v}_{i}^{d}\left(t+1\right)={W}^{d}(t)\times {v}_{i}^{d}\left(t\right)+ {a}_{i}^{d}\left(t\right)$$

19 $${x}_{i}^{d}\left(t+1\right)={x}_{i}^{d}\left(t\right)+{v}_{i}^{d}(t+1)\times c(i)$$

20 $${W}^{d}\left(t\right)={\omega }_{max}-\frac{{\omega }_{max}-{\omega }_{min}}{T}\times t,$$

where ${\omega}_{\text{max}}$ and ${\omega}_{\text{min}}$ are the inertia weights (${\omega}_{\text{max}}$=0.7, ${\omega}_{\text{min}}$=0.1, which is mainly based on experience), and *c*(*i*) is a chaotic sequence. A larger inertia weight can improve the exploration ability, and a smaller inertia weight can improve the mining ability. Moreover, the ergodicity and dynamics of the chaotic sequence have the ability to jump out of the local optimum.

### Statistical analysis

Statistical analysis was performed using MedCalc version 13.0.0.0 (MedCalc software, Ostend, Belgium). The derived parameters are expressed as the mean ‍ ± ‍ standard deviation. The Student’s t-test was used to compare the estimated parameters between HCCs and background liver tissues. The box plot was used to assess the consistency of the estimated *k*_1_ and *k*_3_ for the different methods. The diagnostic performance of *k*_1_ and *k*_3_ among the three methods was compared using receiver operating characteristic (ROC) curve analysis. *P* < 0.05 indicated significant differences. The fitting quality of the TACs among the three methods was compared using the Akaike information criterion (AIC) and the Bayesian information criterion (BIC), and a smaller value represents better curve fitting.

## Results

### Kinetic parameters

 The kinetic parameters (*k*_1_, *k*_2_, *k*_3_ and *k*_4_) and the *HPI* obtained by the three methods are shown in Table 1.

**Table 1:** Parameter estimation results of the three methods

	*k*_1_	*k*_2_	*k*_3_	*k*_4_	*HPI* (%)
NLLS					
HCCs	0.528 ± 0.241	0.535 ± 0.200	0.060 ± 0.054	0.061 ± 0.014	48.8 ± 32.8
Liver tissue	0.362 ± 0.197	0.657 ± 0.195	0.074 ± 0.051	0.018 ± 0.025	10.9 ± 15.7
* P*	0.019	0.066	0.411	< 0.001	< 0.001
GSA					
HCCs	0.679 ± 0.206	0.754 ± 0.183	0.189 ± 0.035	0.111 ± 0.046	56.5 ± 13.8
Liver tissue	0.545 ± 0.073	0.772 ± 0.045	0.161 ± 0.036	0.042 ± 0.034	21.8 ± 7.3
* P*	0.008	0.675	0.019	< 0.001	< 0.001
DCGSA					
HCCs	0.651 ± 0.013	0.592 ± 0.012	0.137 ± 0.024	0.064 ± 0.003	66.7 ± 18.3
Liver tissue	0.628 ± 0.015	0.620 ± 0.013	0.075 ± 0.024	0.090 ± 0.009	31.0 ± 9.2
* P*	< 0.001	< 0.001	< 0.001	< 0.001	< 0.001

NLLS yielded a significant difference in HCCs due to its higher *k*_1_, *k*_4_ and the *HPI* than those in background liver tissue (*P* = 0.019, *P* < 0.001, and *P* < 0.001, respectively), and *k*_2_ and *k*_3_ did not show a significant difference between HCCs and background liver tissue (*P* = 0.067 and *P* = 0.411, respectively).

For the GSA, *k*_1_, *k*_3_, *k*_4_ and the *HPI* showed significant differences in distinguishing between HCCs and background liver tissue (*P* = 0.008, *P* < 0.001, *P* < 0.001, and *P* = 0.019, respectively), while *k*_2_ did not reach significance. (*P* = 0.688).

For the DCGSA, *k*_1_, *k*_3_ and the *HPI* were significantly higher in HCCs than in background liver tissue (*P* < 0.001, *P* < 0.001, and *P* < 0.001, respectively), and *k*_2_ and *k*_4_ were significantly lower in HCCs than in background liver tissue (*P* < 0.001 and *P* < 0.001, respectively).

**Fig. 4:**
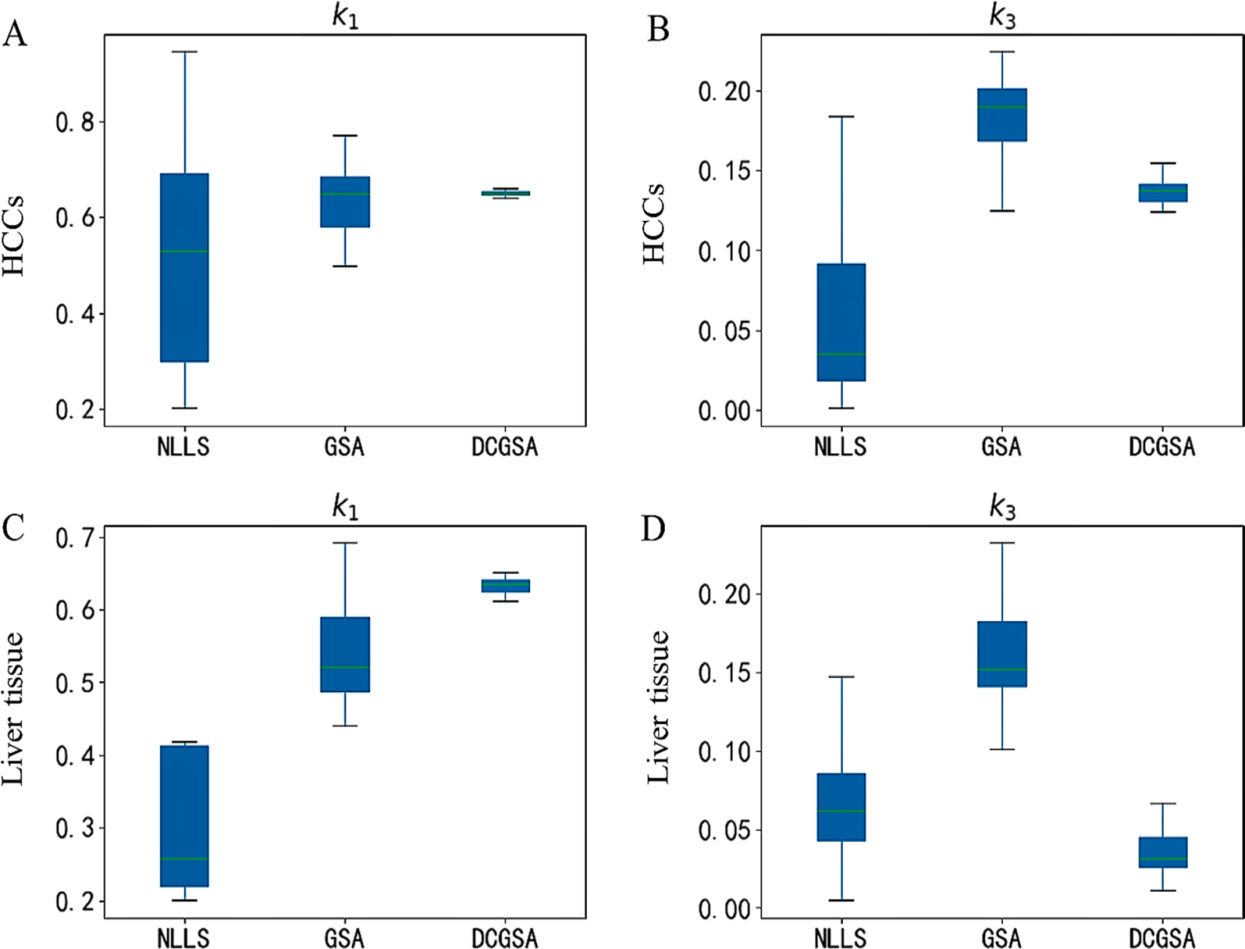
Box plots of *k*_1_ and *k*_3_ for the three methods

The box plots of *k*_1_ and *k*_3_ estimated by the three methods are shown in Fig. 4. Compared with the GSA and NLLS, the DCGSA had a more compact data distribution and lower standard deviation of *k*_1_ and *k*_3_ in HCCs and in background liver tissues.

### Comparison of *k*_1_ and *k*_3_

**Fig. 5:**
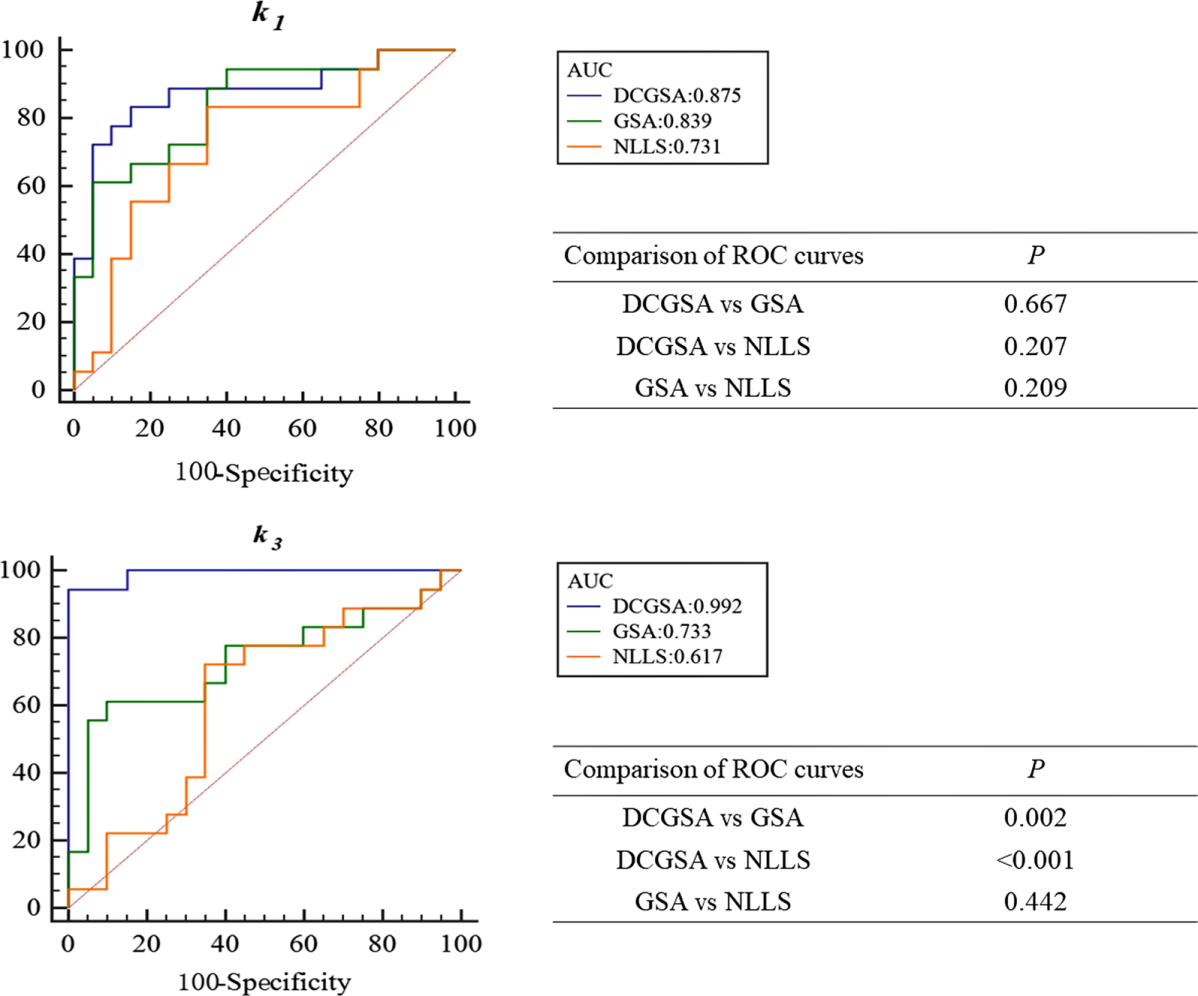
Comparison of the ROC curves of *k*_1_ and *k*_3_

Figure 5 shows the ROC curves of *k*_1_ and *k*_3_ estimated by the three methods. For *k*_1_, the diagnostic performance for differentiating HCCs from background liver tissue among the three methods was not significantly different (all *P* > 0.05). For *k*_3_, the DCGSA had higher diagnostic performance than the GSA and NLLS (*P* = 0.0024 and *P* = 0.0001, respectively); the diagnostic performance was not significantly different between the GSA and NLLS (*P* = 0.4420).

### TAC fit quality

**Fig. 6:**
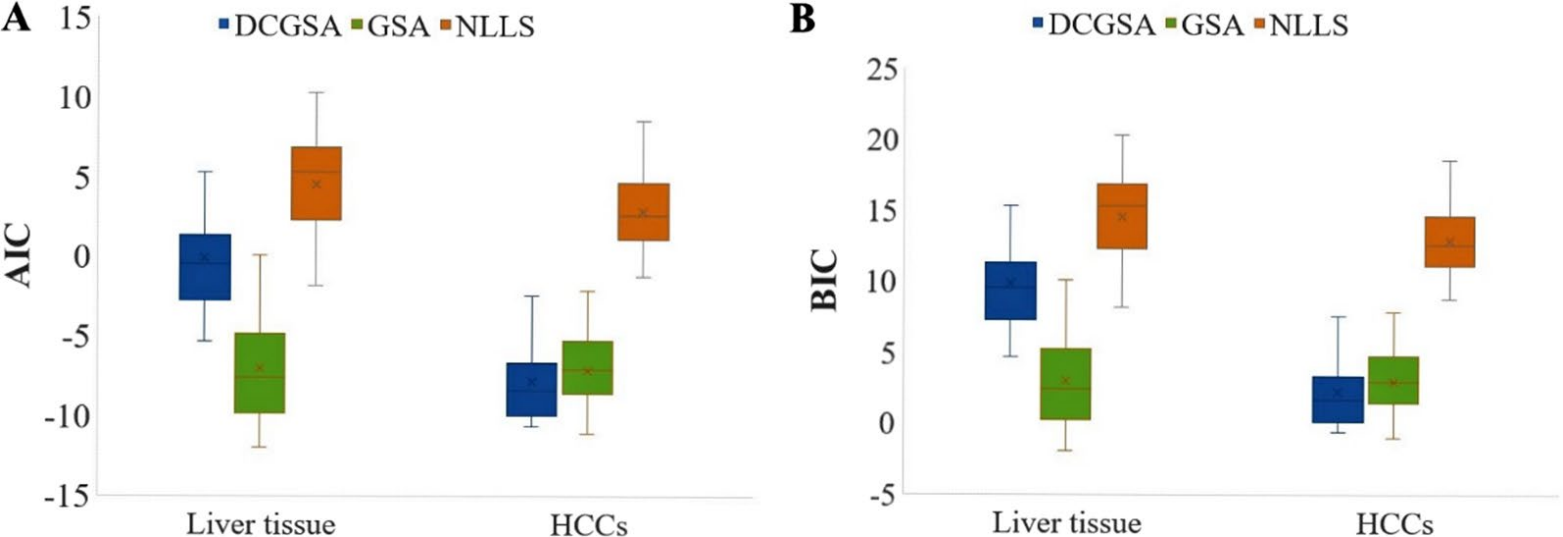
AIC and BIC values of TAC fitting by the three methods

Figure 6 shows the mean and standard deviation of the AIC and BIC values for HCCs and background liver tissue using the DCGSA, GSA and NLLS. The DCGSA had the lowest AIC and BIC values among the three methods for HCCs, and the AIC and BIC values of the DCGSA were higher than those of the GSA and lower than those of NLLS for the background liver tissue.

## Discussion

Conventional dynamic scans take 60 min or more, which is time-consuming, significantly limits the daily throughput of PET/CT scanners and the patients cannot remain immobile for a long period of time, thus it is not suitable for clinical settings. Based on previous study results, this paper used 5-min dynamic PET/CT for kinetic analysis to differentiate HCCs from background liver tissue [11, 12]. This study preliminarily introduced the GSA and improved kinetic parameter estimation with a dual-input dual-compartment model on dynamic PET/CT. The liver receives dual blood supplies from the hepatic artery and portal vein, which is neglected in traditional single-input kinetic modeling, resulting in inaccuracy in kinetic parameter estimation [25, 26]. Moreover, hepatocytes contain glucose-6-phosphatase (G6P), which dephosphorylates ^18^FDG-6-phosphate (*k*_4_) [6]. In this paper, a reversible (*k*_4_ > 0) two-tissue three compartment model with dual blood input is used for kinetic modeling, and it can accurately describe the glucose metabolism of the liver.

With the development of swarm intelligence optimization algorithms, some researchers have begun to use them to estimate kinetic models. Liu et al. [27] proposed an improved artificial immune network (AIN) for parameter estimation of a normal mouse liver kinetic model. Huang et al. [28] achieved kinetic analysis of PET images of Parkinson’s disease by particle swarm optimization (PSO). Sara et al. [29] also demonstrated that the kinetic modeling of mouse kidneys can be estimated by ant colony optimization (ACO). The GSA is an optimization algorithm inspired by the theory of Newtonian gravity; its role in kinetic parameter estimation has attracted the attention of researchers. Ismail et al. [30] proposed a hybrid algorithm of PSO and the GSA for kinetic model parameter estimation of aspartate biochemical pathways.

This study preliminarily indicated that the GSA can estimate the parameters of the liver kinetic model and can distinguish HCCs from background liver tissue by *k*_1_, *k*_3_, *k*_4_ and the *HPI*. To further enhance the global search capability and improve the strategy for jumping out of the local optimum to improve the accuracy of parameter estimation, an enhanced version, the DCGSA, is proposed in this paper, which showed significant differences in the kinetic parameters *k*_1_, *k*_2_, *k*_3_, and *k*_4_ and the *HPI*. NLLS is the most commonly used parameter estimation of kinetic models, but it can only distinguish HCCs from background liver tissue by *k*_1_, *k*_4_ and the *HPI*. Additionally, the statistical information criteria showed that the DCGSA achieved lower AIC and BIC values than those of the GSA and NLLS for HCCs and lower AIC and BIC values than those of NLLS for background liver tissue, which ensures improved TAC fitting quality.

Normal liver tissue is mainly supplied by the portal vein, which carries 70%-80% of the overall inflow, while HCC is a hypervascular tumor mainly supplied by the hepatic artery. The results of this study are consistent with those of previous studies, with the *HPI* of HCC being significantly higher than that of normal liver tissue [10, 12]. The *HPIs* of HCC for NLLS, the GSA, and the DCGSA were 48.8 ± 32.8, 56.5 ± 13.8, and 66.7 ± 18.3, respectively, and the DCGSA was much closer to the expected clinical value. Most patients with HCC have a history of liver cirrhosis, which leads to an increase in the arterial blood supply of normal liver tissue [31]. When using the GSA and NLLS, the respective *HPIs* of normal liver tissue were 10.9 ± 15.7 and 21.8 ± 7.3 and were much lower than expected. However, the *HPI* of the DCGSA was 31.0 ± 9.2 and is closer to clinical practice.

Many glucose transporters (Gluts), which transport ^18^F-FDG from the blood into hepatocytes, are distributed on the plasma membrane of hepatocytes, and ^18^F-FDG is phosphorylated to ^18^F-FDG-6-phosphate by hexokinase (HK). The expression of Gluts is significantly higher in many cancer cells than in normal cells, and the uptake of glucose is increased. In addition, the expression of hexokinase and its affinity or functional activity of glucose phosphorylation is generally higher in cancer cells [32–35]. The transport rate *k*_1_ and phosphorylation rate *k*_3_, which are the parameters of major interest, are often used for quantitative analysis to achieve the diagnosis and assessment of HCC. Zuo et al. [36] reported the potential of using *k*_1_ to noninvasively evaluate human liver inflammation. Geist et al. [13] compared four different kinetic models for kinetic modeling of HCC and demonstrated significant differences in *k*_3_ between HCC and background liver tissue in all models.

In this study, *k*_1_ and *k*_3_ were greater in HCC than in background liver tissue by using the GSA and DCGSA, which is consistent with previous studies, implying increased Glut and HK activity in HCC [10, 37]. Meanwhile, the box plots showed higher consistency of *k*_1_ and *k*_3_ estimated with the DCGSA compared with NLLS and the GSA. Comparison of the ROC curves showed that the DCGSA had the highest diagnostic performance for *k*_3_ and was significantly higher than that of the GSA and NLLS.

G6P activity and the dephosphorylation rate *k*_4_ are higher in normal liver tissue than in HCC [6]. Our results showed that only *k*_4_ of the DCGSA conformed to the above clinical study, but that of the GSA and NLLS did not.

In summary, ^18^F-FDG kinetics can provide a comprehensive understanding of physiological systems and disease pathogenesis. The DCGSA can better estimate the kinetic parameters of the compartment model than the GSA and NLLS, which might play a significant role in clinical use for tumor characterization, monitoring the locoregional therapy and outcomes after resection.

This study has some limitations. First, the sample size of the experimental data is small. Second, there was a slight difference in ROI placement during TAC extraction, but we believe the influence was negligible. Third, this study did not explore lymph node involvement and other sites of metastasis, and we may explore other kinetic models in further studies. Fourth, the parameter estimation process is computationally expensive, and the proposed DCGSA might not be applicable to voxel level analysis for parametric images. It is necessary to improve the fitting algorithm to enhance the diagnostic performance and to develop more effective and efficient methods in our future research.

## Conclusions

In this study, we demonstrated that the GSA can be used for parameter estimation of kinetic models on dynamic ^18^F-FDG PET/CT, and furthermore, the DCGSA was proposed to estimate the parameters more efficiently and reliably to distinguish HCC from background liver tissue.

## Data Availability

The datasets generated and analyzed during the current study are not publicly available due to the security of data but are available from the corresponding author upon reasonable request.

## Abbreviations

PET: Positron emission tomography
CT: Computed tomography
MRI: Magnetic resonance imaging
^18^F-FDG: Fluorine-18-fluorodeoxyglucose
HCC: Hepatocellular carcinoma
DCGSA: Dynamic chaotic gravitational search algorithm
GSA: Gravitational search algorithm
NLLS: Nonlinear least squares
HPI: Hepatic arterial perfusion index
SUV: Standardized uptake value
ROI: Regions of interest
TAC: Time-activity curve
OSEM: Ordered subsets expectation maximization
AIC: Akaike information criterion
BIC: Bayesian information criterion
AIN: Artificial immune network
PSO: Particle swarm optimization
ACO: Ant colony optimization
Gluts: Glucose transporters
HK: Hexokinase
G6P: Glucose-6-phosphatase
